# Development of a prediction model for postoperative urinary tract infection in ureteral stone patients based on automated machine learning models

**DOI:** 10.3389/fphys.2026.1768212

**Published:** 2026-05-26

**Authors:** Ding Li, Tiantian Fang, Shaojiang Li

**Affiliations:** Department of Urology, Chun’an County First People’s Hospital, Hangzhou, China

**Keywords:** automated machine learning (AutoML), clinical decision support system, explainable artificial intelligence, postoperative urinary tract infection (PO-UTI), ureteral stone

## Abstract

**Objective:**

This study aimed to develop a predictive model for postoperative urinary tract infection (PO-UTI) in ureteral stone patients, addressing limitations of traditional research methods and advancing perioperative infection management from experience-driven to data-driven transformation.

**Methods:**

A retrospective cohort design was employed, enrolling 826 ureteral stone surgery patients (January 2020 to January 2024) with data on demographics, disease characteristics, and hematological indicators collected via structured electronic medical records. Feature selection was optimized using an improved ISequoiaOA meta-heuristic algorithm to enhance model optimization stability; the SMOTE-ENN hybrid sampling technique was applied to balance class distribution; an AutoML framework integrating SHAP interpretability analysis was constructed to quantify feature contribution and visualize interaction effects; and a clinical decision support system was developed.

**Results:**

(1) optimal performance of the AutoML model on the test set (ROC-AUC=0.9251, PR-AUC=0.8712), significantly outperforming traditional algorithms such as XGBoost and LightGBM; (2) key predictors identified via SHAP analysis included preoperative urinary retention, low serum albumin (ALB), diabetes, double-J stent indwelling time, postoperative catheter indwelling time, and age, with interaction effects revealing a nonlinear synergistic surge in infection risk when stone size exceeded 6 mm and catheter indwelling time exceeded 5 days.

**Conclusion:**

This study integrated AutoML and explainable AI technologies to construct an accurate PO-UTI prediction model, groundbreakingly quantifying biological thresholds for synergistic effects and providing a “data-mechanism dual-driven” new paradigm for perioperative infection prevention and control.

## 1 Introduction

Urinary tract stones constitute a highly prevalent urological disorder worldwide, with ureteral stones accounting for approximately 65%-70% of upper urinary tract stones (Sunaryo et al., 2022). The prevention and management of complications following surgical intervention remain pivotal yet challenging aspects of urological clinical practice (Johnson et al., 2025). Postoperative urinary tract infection (PO-UTI) is a major complication of common procedures like ureteroscopic lithotripsy, with reported incidence rates ranging from 8.6% to 25.4% (Chi et al., 2025). PO-UTI not only prolongs hospitalization and increases healthcare expenditures but also heightens the risk of severe secondary complications such as sepsis (Wu et al., 2023). Current clinical practice predominantly relies on empirical antibiotic prophylaxis and static risk assessment scales (e.g., Charlson Comorbidity Index) for intervention decisions. However, traditional approaches exhibit significant limitations: firstly, static scales inadequately incorporate dynamic perioperative indicators, resulting in constrained efficacy for time-sensitive postoperative infection prediction; secondly, logistic regression-based prediction models face modeling bottlenecks when handling high-dimensional, nonlinear clinical data (Bertsimas et al., 2021; Zhuang et al., 2024).

Recent breakthroughs in machine learning (ML) technology have provided a new paradigm for clinical prediction model development (Kanwal et al., 2020). As a core branch of artificial intelligence, ML enables computer systems to autonomously discover patterns and generate predictive rules from data, overcoming traditional statistical models’ dependency on predefined functional forms (Kanwal et al., 2020). Notably, the emergence of automated machine learning (AutoML) frameworks has substantially lowered modeling barriers by automating the entire workflow from feature preprocessing and algorithm selection to hyperparameter optimization, addressing the pain point of extensive manual parameter tuning in traditional modeling (Qu et al., 2025). In urology, studies have demonstrated that ensemble learning models based on XGBoost and LightGBM outperform conventional statistical methods in predicting stone recurrence (Khene et al., 2023; Chen et al., 2024). Nevertheless, applying AutoML to PO-UTI prediction faces three critical challenges: inherent class imbalance in clinical data predisposing models to bias toward majority classes; conventional feature selection methods struggling to capture interaction effects among high-dimensional features; and insufficient model interpretability hindering clinical translation.

In-depth analysis of existing literature reveals three major limitations (Khan et al., 2022; Choi et al., 2024; Favresse et al., 2025; Zhang et al., 2025): (1) Inadequate sample representativeness, as most studies fail to rigorously exclude confounding factors like immunodeficiency; (2) Suboptimal model optimization due to unaddressed severe imbalance in medical data, where previously validated techniques such as SMOTE (employed in this study) remain unreported for PO-UTI prediction; and (3) Deficiency in clinical application, where current achievements rarely integrate implementable decision support systems, particularly lacking tools for visual interpretation of predictive logic. Crucially, no existing research systematically explores metaheuristic algorithms’ potential for clinical feature optimization, indicating continued reliance on empirical knowledge rather than data-driven methods for identifying key biomarkers during model construction.

Based on these considerations, this study aims to overcome traditional PO-UTI prediction limitations through AutoML technology, advancing perioperative management from experience-driven to data-driven transformation and establishing a new paradigm for precision infection prevention in urology.

## 2 Methods

### 2.1 Study population

This retrospective cohort study enrolled 826 ureteral stone patients admitted to Chun’an County First People’s Hospital from January 2020 to January 2024. Approval was obtained from the hospital’s Ethics Committee (Approval No.: 2025041217) in accordance with the Declaration of Helsinki, with informed consent waived due to the retrospective design.

Inclusion criteria: (1) Diagnosed with ureteral stones based on standard criteria (Skolarikos et al., 2025); (2) Underwent surgical intervention; (3) Complete medical records available.

Exclusion criteria: (1) Immunocompromised status; (2) Concurrent urinary tract disorders; (3) Severe organ dysfunction.

### 2.2 Data collection

Data were extracted from structured electronic medical records by two certified researchers. Variables included: (1) Demographics: Sex, age, body mass index (BMI), hypertension, diabetes; (2) Disease characteristics: Stone duration, stone location, stone size, preoperative urinary retention; (3) Perioperative indicators: Operation duration, postoperative catheter indwelling time, double-J stent indwelling time, hospital stay, hydronephrosis; (4) Hematological indicators: White blood cell count (WBC) and serum albumin (ALB) measured within 48 hours postoperatively; (5) Outcome: PO-UTI diagnosis within 24 hours post-surgery, defined per EAU Guidelines on Urolithiasis (Skolarikos et al., 2025).

During the data preprocessing phase, we systematically evaluated the missingness of all included variables. The clinical variables retrospectively collected in this study consisted primarily of mandatory fields or routine test items from structured electronic medical records, yielding a low overall missing rate (<5%). Specifically, missing values were concentrated in laboratory indicators measured within 48 hours postoperatively (e.g., ALB missing rate 2.3%; WBC missing rate 1.8%), attributable to reasons such as early patient discharge or unfulfilled test orders. To avoid potential information loss and selection bias, we refrained from applying simple complete-case analysis for handling missing data. Instead, we implemented multiple imputation by chained equations (MICE), a more robust processing strategy. The specific workflow proceeded as follows: First, the mice package in R software (version 4.3.1) was utilized to construct the imputation model. This model incorporated all features relevant to variables with missing data [including demographics, disease characteristics, surgical variables, and the outcome variable (PO-UTI) to ensure conditional randomness during imputation. We generated five independent imputed datasets, with the iteration count for each imputation set to 10. For continuous variables (e.g., ALB, WBC), predictive mean matching was employed, while logistic regression was adopted for imputing categorical variables. Subsequently, all steps concerning model development, feature selection, and performance evaluation were conducted independently on each imputed dataset. The final model performance metrics (e.g., AUC, accuracy) were pooled across the five imputed datasets using Rubin’s rules, yielding overall estimates that accounted for imputation uncertainty. This approach maximally preserved the sample size, effectively minimized potential bias introduced by missing data, and ensured model construction based on a complete and consistent dataset.

### 2.3 Model development

Using stratified random sampling, the dataset was split into training (n=661, 80%) and test sets (n=165, 20%) based on outcome distribution. An AutoML framework embedding an Improved Sequoia Optimization Algorithm (ISequoiaOA) was proposed to simultaneously optimize feature selection and hyperparameter tuning. The ISequoiaOA enhanced the original SequoiaOA (a metaheuristic algorithm inspired by redwood forest self-regulation dynamics) via chaotic mapping for population initialization and dynamic Lévy flight step size to balance exploration-exploitation efficiency. For reproducibility, the key implementation details of ISequoiaOA are as follows: (1) Parameter Settings: The algorithm’s search space for hyperparameter optimization was defined based on the candidate models, regularization parameters: [0.01, 10]). The population size was set to 30, and the maximum number of iterations was 500. The chaotic mapping utilized a logistic map with an initial value of 0.7, while the Lévy flight step size parameter (β) was dynamically adjusted between 1.0 and 1.5 based on iteration progress. (2) Convergence Criterion: The optimization process terminated if the global best fitness value (maximizing the ROC-AUC on the validation set) showed no improvement (Δfitness< 1e-5) for 50 consecutive generations, or when the maximum iteration count was reached.

The framework employed a two-phase optimization: Phase 1: Discrete space feature subset selection. Phase 2: Continuous space hyperparameter fine-tuning. Six comparative models were implemented in MATLAB 2024b: Logistic Regression (LR), Support Vector Machine (SVM), AdaBoost, XGBoost, LightGBM, and the proposed AutoML framework. The selection of these comparative models was motivated by the need to benchmark the proposed AutoML framework against a spectrum of established and clinically relevant algorithms. Logistic Regression (LR) was included as a fundamental statistical baseline, representing interpretable, linear models commonly used in clinical practice. Support Vector Machine (SVM) served as a representative of classical, kernel-based machine learning methods with robust performance in high-dimensional spaces. Adaboost was chosen as an early and influential ensemble learning algorithm, providing a benchmark for boosting techniques. XGBoost and LightGBM were selected as state-of-the-art, gradient-boosting ensemble models that have consistently demonstrated superior predictive accuracy in recent biomedical informatics studies and thus represent a high-performance benchmark. Comparing against this gradient of models—from traditional statistics to modern complex ensembles—allows for a comprehensive evaluation of whether the proposed AutoML framework offers tangible improvements beyond both common clinical tools and cutting-edge manual ML implementations.

**Figure 1:**
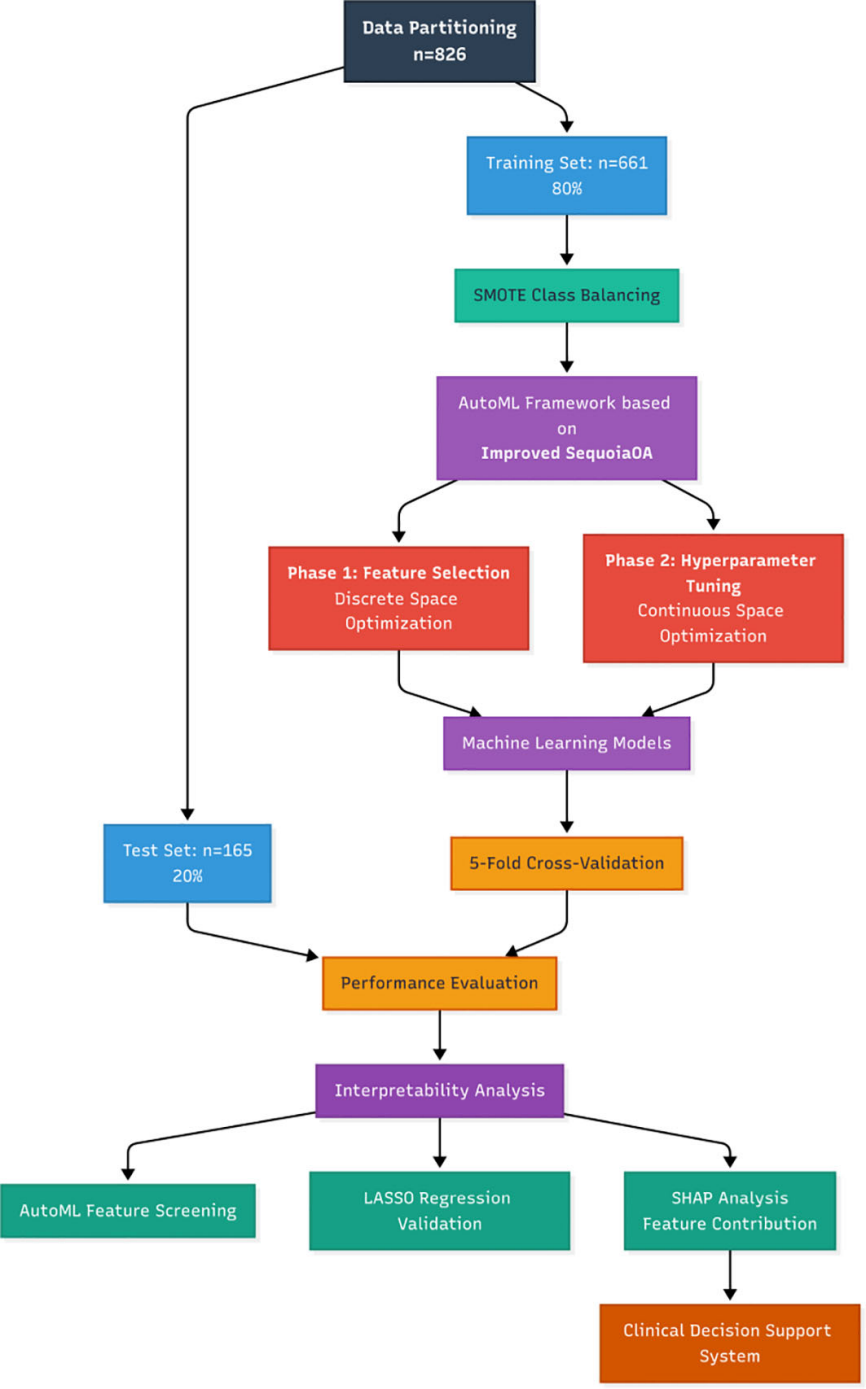
Study flowchart.

All models used five-fold cross-validation after standardization and Synthetic Minority Oversampling Technique (SMOTE) for class imbalance mitigation (**Figure 1**). To provide a clearer illustration of the AutoML framework’s workflow as a complement to **Figure 1** (Study Flowchart), pseudocode detailing its core two-phase optimization process is presented in **Supplementary Material 1**.

### 2.4 Evaluation metrics

Model performance was assessed through: (1) Classification accuracy: ROC-AUC, Precision-Recall AUC (PR-AUC), Accuracy, Sensitivity, Specificity, Precision, and F1-score; (2) Calibration performance: Calibration curves and Brier score; (3) Clinical utility: Decision Curve Analysis (DCA) calculating net benefit (NB):


$$NB=\frac{TP}{N}-\frac{FP}{N}\times \frac{\rho_{t}}{1-\rho_{t}}$$

where *TP* = true positives, *FP* = false positives, *N* = total samples, and *p_t_* = risk threshold. *NB* was compared against standard intervention strategies to identify clinically effective thresholds.

### 2.5 Interpretability analysis

Following preliminary screening of prognostic predictive features using the AutoML framework, LASSO regression analysis was further employed to validate the robustness of the selected features. Finally, the SHAP (Shapley Additive exPlanations) interpretability model was utilized to analyze the clinical rationality of the features. The specific workflow is as follows: (1) AutoML primary feature screening: Leveraging AutoML algorithms, significant feature subsets related to prognosis were automatically identified based on predefined search spaces and optimization objectives; (2) LASSO feature validation: LASSO regression was applied to the feature subsets screened by AutoML, verifying their sparsity and stability through regularization constraint mechanisms to ensure key features’ anti-overfitting capability. A comparison of differences between LASSO-selected and AutoML-automatically selected features was conducted; (3) SHAP interpretability analysis: Based on game theory, the SHAP algorithm quantified feature contributions. Global feature importance ranking revealed the overall impact intensity of key variables, enabling visualization of prediction logic to subsequently validate rationality. SHAP analysis quantified predictive contribution weights for key biomarkers without implying biological causality, as feature importance reflects statistical association within the cohort.

### 2.6 Clinical decision support system

In our study, MATLAB’s App Designer functionality was utilized to develop a clinical decision support software. This software integrates the constructed predictive model, aiming to provide clinicians with an intuitive and user-friendly tool for assessing patient prognosis. The software can be deployed to the Web for clinical convenience.

### 2.7 Statistical methods

Research data were uniformly imported into the SPSS 26.0 statistical analysis platform for standardized processing. Continuous variables conforming to normal distribution were expressed as mean ± standard deviation (x̄ ± s), while non-normally distributed continuous variables were expressed as median (interquartile range) (M [IQR]). Categorical variables were expressed as frequencies and percentages [n(%)]. For between-group comparisons: Continuous variables: Normality testing was first performed. If both groups satisfied normal distribution, an independent samples t-test was used; if not, the Mann-Whitney U test was applied. Categorical variables: Pearson’s chi-square test was employed for between-group comparisons. In addition to descriptive statistics and cross-validation, statistical comparisons of model performance were conducted. The DeLong test, a non-parametric method for comparing the areas under two correlated receiver operating characteristic (ROC) curves derived from the same sample, was employed to assess the significance of differences in ROC-AUC between the proposed AutoML model and each baseline model on both the training (via cross-validation) and independent test sets. A two-sided p-value of less than 0.05 was considered statistically significant. All statistical tests were performed using R software (version 4.3.1) with the pROC package.

## 3 Results

### 3.1 Patient characteristics

This study enrolled 826 patients, of whom 236 developed postoperative urinary tract infections, yielding an infection rate of 28.57%. A total of 258 pathogenic strains were isolated from these 236 PO-UTI patients, predominantly *Escherichia coli*, *Pseudomonas aeruginosa*, *Staphylococcus aureus*, *Streptococcus haemolyticus*, and *Candida albicans* (**Table 1**). The dataset was split into training (n=661) and test sets (n=165) at an 8:2 ratio. No statistically significant differences existed in baseline characteristics between the two sets (P>0.05) (**Table 2**). The training set contained 189 positive cases, while the test set had 47 positive cases, confirming the validity of stratified random sampling (28.59% vs. 28.48%; χ²=0.001, P = 0.978).

**Table 1:** Pathogen distribution.

Pathogen	Strains (n=258)	Proportion (%)
Gram-negative bacteria	152	58.91
*Escherichia coli*	48	18.60
*Pseudomonas aeruginosa*	42	16.28
*Proteus*	24	9.30
*Klebsiella pneumoniae*	20	7.75
*Enterobacter cloacae*	12	4.65
Others	6	2.33
Gram-positive bacteria	96	37.21
*Staphylococcus aureus*	36	13.95
*Streptococcus haemolyticus*	26	10.08
*Staphylococcus epidermidis*	18	6.98
*Enterococcus faecalis*	12	4.65
Others	4	1.55
Fungi (Candida albicans)	10	3.88

**Table 2:** Comparison of patient characteristics between datasets.

Variable	Training set (n=661)	Test set (n=165)	Statistic	*P*-value
Age (years)	52.3 ± 10.1	51.8 ± 9.6	t = 0.58	0.562
Sex (Male)	382 (57.9%)	92 (55.8%)	χ² = 0.17	0.687
BMI (kg/m^2^)	24.2 ± 3.1	23.9 ± 3.3	t = 1.01	0.312
Hypertension (Yes)	198 (30.0%)	52 (31.5%)	χ² = 0.10	0.756
Diabetes (Yes)	115 (17.4%)	27 (16.4%)	χ² = 0.06	0.813
Stone Duration				
<1month	431 (65.2%)	106 (64.2%)	χ² = 0.05	0.831
≥1month	230 (34.8%)	59 (35.8%)	χ² = 0.05	0.831
Stone Location				
Upper ureter	495 (74.9%)	122 (73.9%)	χ² = 0.04	0.845
Mid/Lower ureter	166 (25.1%)	43 (26.1%)	χ² = 0.04	0.845
Stone size (mm)	12.1 ± 1.7	12.3 ± 1.6	t = 1.22	0.223
Preoperative retention (Yes)	153 (23.2%)	36 (21.8%)	χ² = 0.12	0.729
Operative time (min)	70.2 ± 12.3	69.8 ± 11.9	t = 0.35	0.727
Catheter duration (d)	2.0 [1.0–3.0]	2.0 [1.0–3.0]	U = 53900	0.812
Double-J stent duration (weeks)	5.0 ± 1.0	4.9 ± 1.1	t = 0.92	0.358
Hospital stay (d)	7.0 [6.0–8.0]	7.0 [6.0–8.0]	U = 54120	0.786
Hydronephrosis (Yes)	561 (84.9%)	139 (84.2%)	χ² = 0.03	0.862
WBC(×10^9^/L)	10.9 ± 2.1	10.7 ± 2.0	t = 1.07	0.285
ALB (g/L)	45.8 ± 3.2	45.5 ± 3.4	t = 0.98	0.327

### 3.2 Algorithm improvement performance

**Figure 2:**
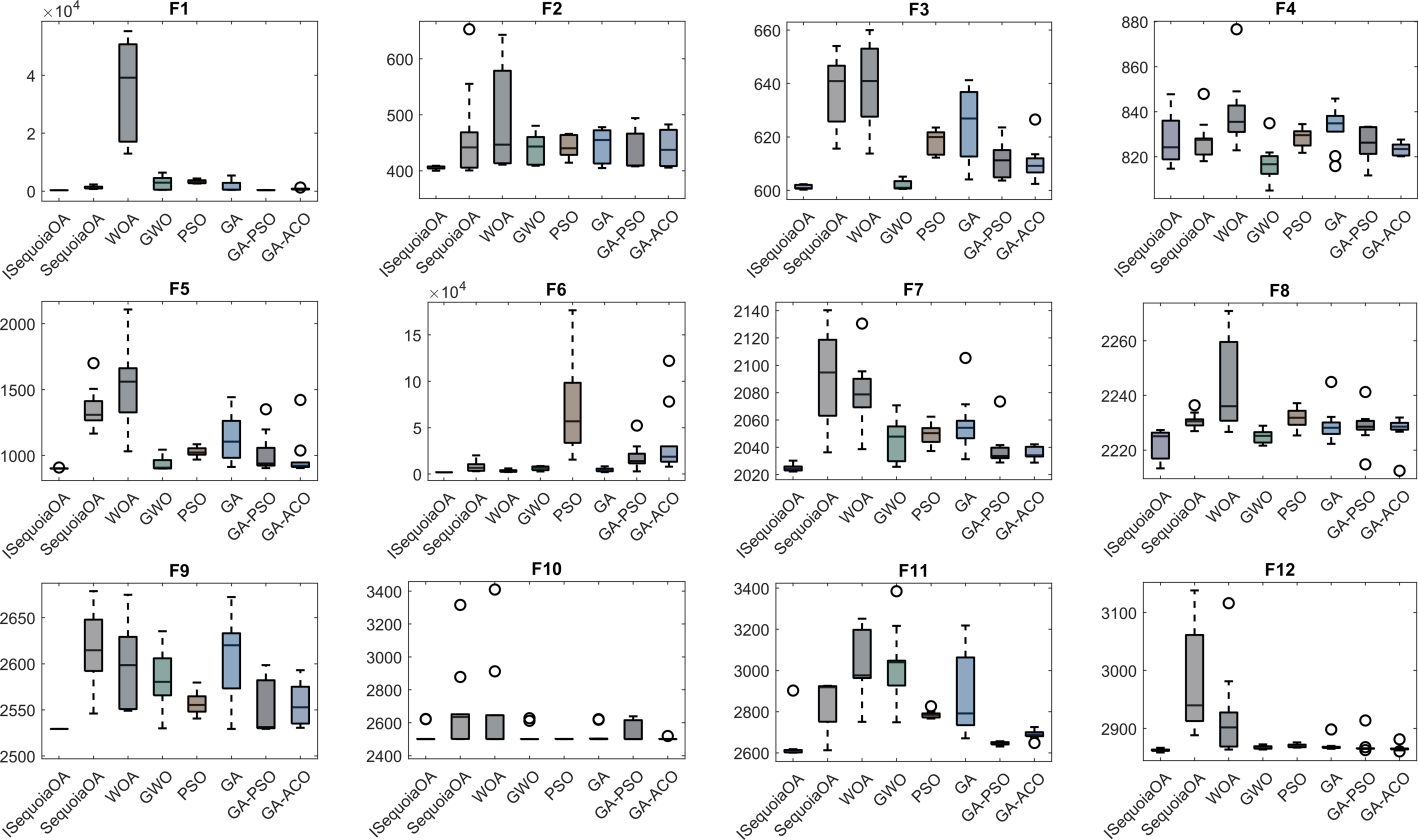
Comparison of optimization performance for swarm intelligence algorithms. Note: Comparison of solution stability across different algorithms on each test function. This panel consists of 12 subplots corresponding to the 12 CEC2022 benchmark functions (F1–F12). In each subplot, the x-axis represents the eight algorithms compared (ISequoiaOA, SequoiaOA, WOA, GWO, PSO, GA, GA-PSO, GA-ACO), and the y-axis represents the best fitness value (optimal solution) obtained from 30 independent runs. The box plots illustrate the distribution and stability of the solutions obtained by each algorithm on a specific function.

**Figure 3:**
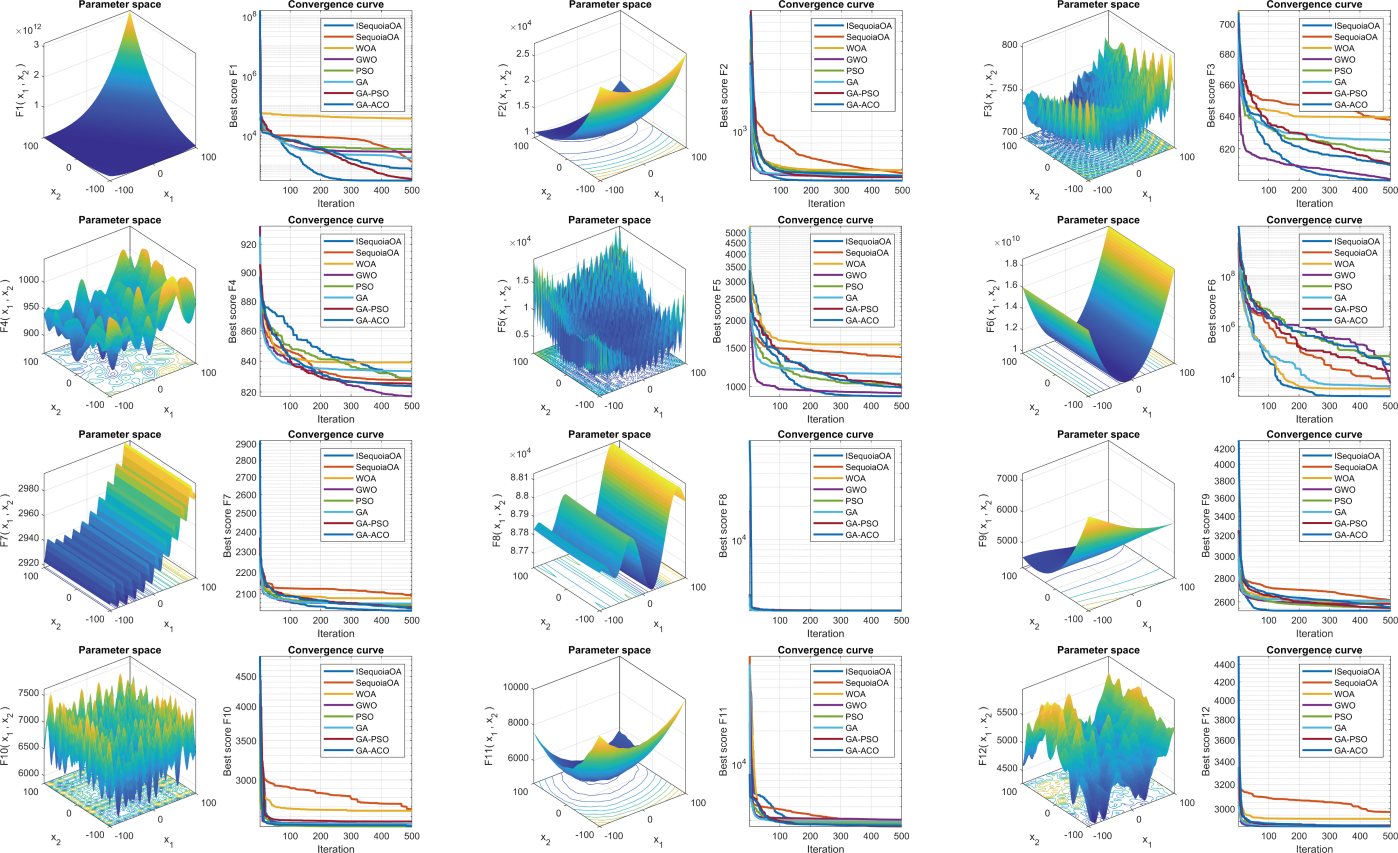
Comparison of convergence performance for swarm intelligence algorithms. Note: Comparison of convergence curves across different algorithms on each test function. This panel consists of 12 subplots corresponding to the 12 CEC2022 benchmark functions (F1–F12). In each subplot, the x-axis represents the iteration number (1–500), and the y-axis represents the best fitness value of the current population (logarithmic scale). Each curve depicts the evolution of the best solution found by an algorithm as the iterations proceed. Experimental settings: All tests were conducted with a dimension of 10, a population size of 30, and a maximum of 500 iterations. The results shown are statistical summaries from 30 independent runs.

To evaluate ISequoiaOA’s optimization capability, we compared it with original SequoiaOA, Whale Optimization Algorithm (WOA), Grey Wolf Optimizer (GWO), Particle Swarm Optimization (PSO), Genetic Algorithm (GA), Genetic Algorithm-Particle Swarm Optimization (GA-PSO), and Genetic Algorithm-Ant Colony Optimization (GA-ACO) using all 12 CEC2022 benchmark functions. Parameters: dimension=10, population size=30, max iterations=500, with 30 independent runs. Boxplots demonstrated ISequoiaOA’s superior stability across most functions (**Figure 2**). Convergence curves confirmed ISequoiaOA’s faster convergence rate and lower risk of local optima (**Figure 3**).

### 3.3 Model training results

**Figure 4:**
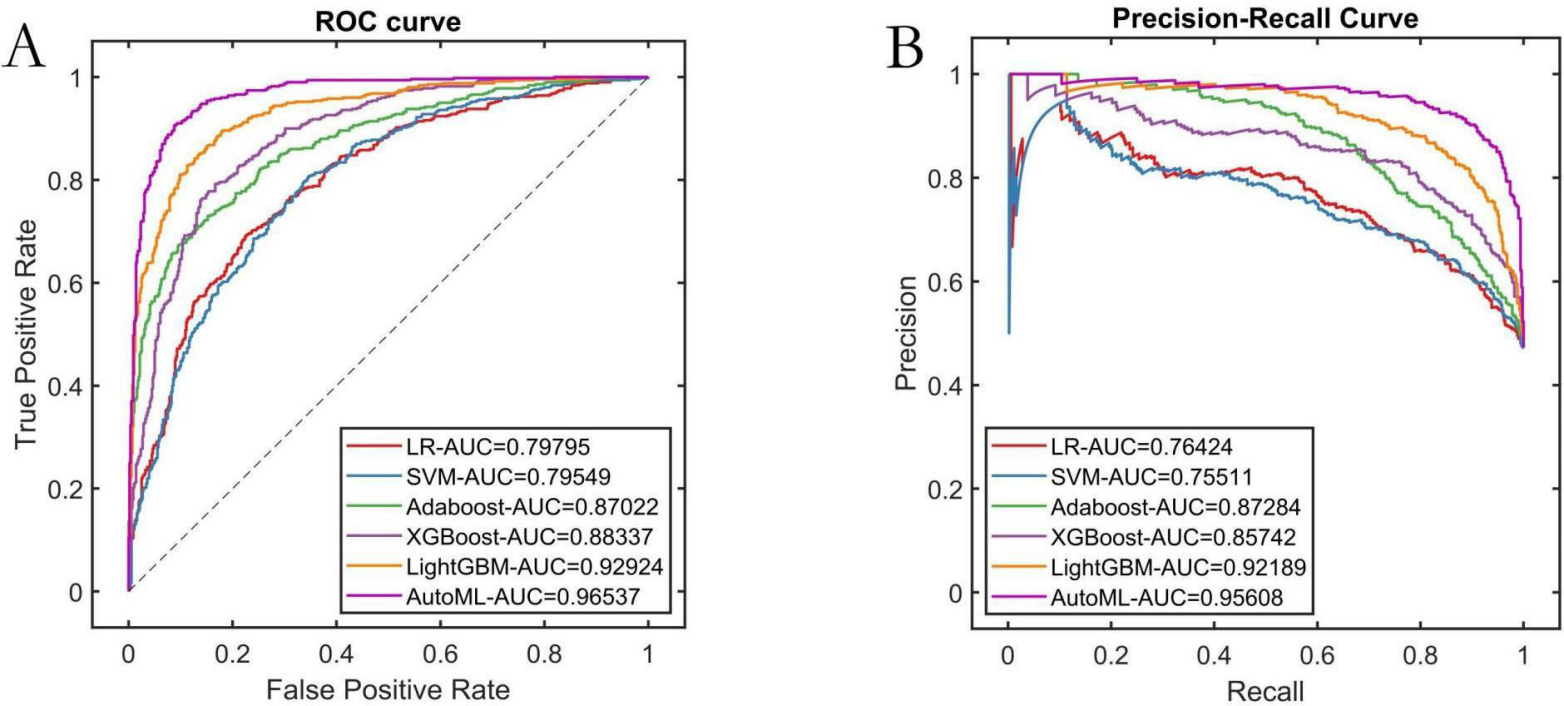
Cross-validation performance on the training set. Note: **(A)** ROC curve of the training set; **(B)** PR curve of the training set.

The AutoML model achieved optimal performance on the training set: ROC-AUC=0.9654, PR-AUC=0.9561, F1-score=0.8561 (**Table 3**; **Figures 4A, B**). Key features selected: Age, Diabetes, Preoperative retention, Catheter duration, Double-J stent duration, ALB.

**Table 3:** Training set cross-validation performance.

Models	PRE	SEN	SPE	ACC	F1	ROC-AUC	PR-AUC
LR	0.4896	0.9900	0.0753	0.5076	0.6552	0.7980	0.7642
SVM	0.6411	0.8540	0.5717	0.7051	0.7324	0.7955	0.7551
Adaboost	0.5814	0.9500	0.3871	0.6531	0.7213	0.8702	0.8728
XGBoost	0.6236	0.9740	0.4731	0.7098	0.7603	0.8834	0.8574
LightGBM	0.6112	0.9840	0.4391	0.6966	0.7540	0.9292	0.9219
AutoML	0.7589	0.9820	0.7204	0.8440	0.8561	0.9654	0.9561

### 3.4 Predictive performance on test set

**Figure 5:**
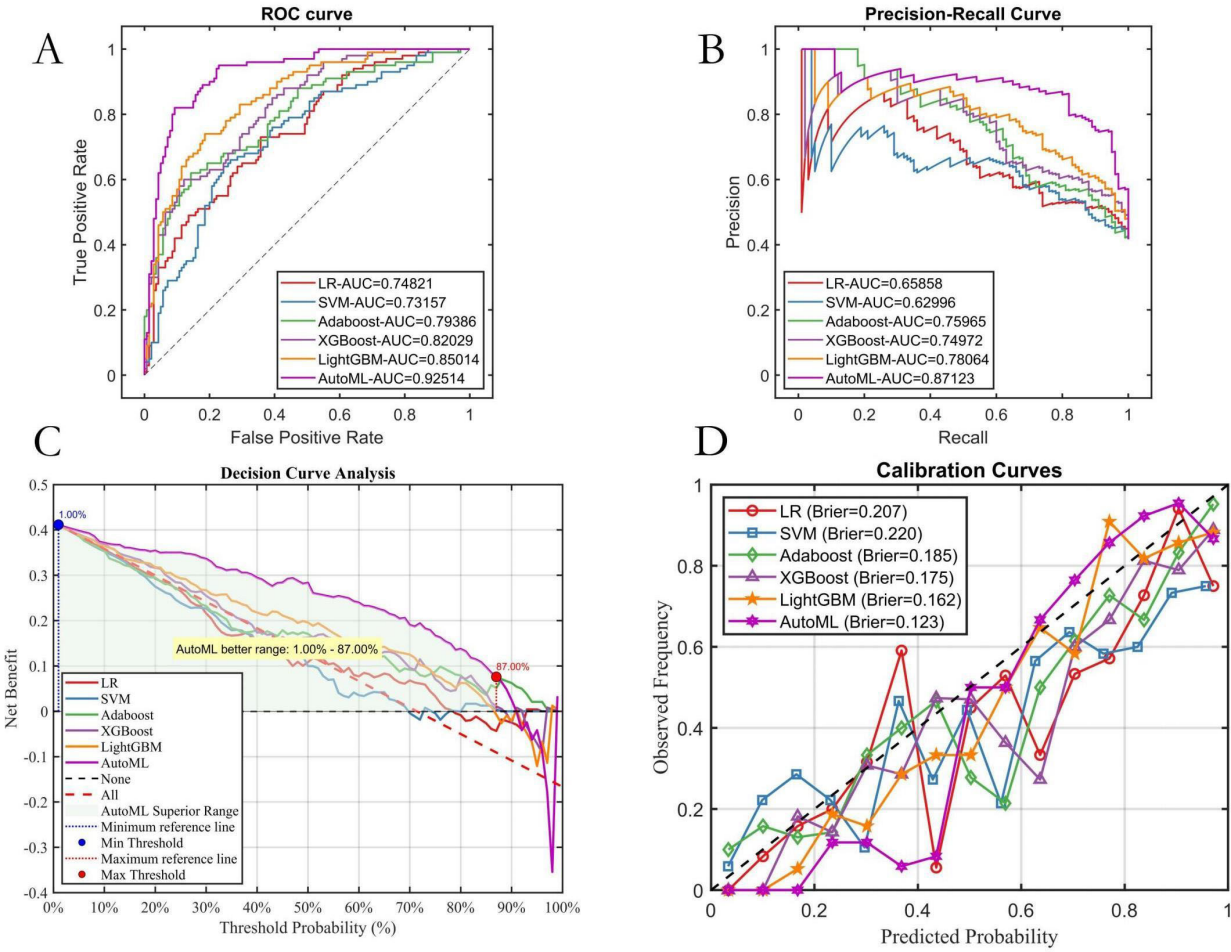
Predictive performance on the test set. Note: **(A)** ROC curve of the test set; **(B)** PR curve of the test set; **(C)** DCA curve of the test set; **(D)** Calibration curve of the test set.

AutoML showed robust performance: ROC-AUC=0.9251, PR-AUC=0.8712 (**Figures 5A, B**; **Table 4**). Decision Curve Analysis (DCA) indicated greater net benefit at 1%~87% risk thresholds vs. traditional methods (**Figure 5C**). Calibration curves confirmed AutoML’s superior accuracy (Brier score=0.123) (**Figure 5D**).

**Table 4:** Test set performance.

Models	PRE	SEN	SPE	ACC	F1	ROC-AUC	PR-AUC
LR	0.4167	1.0000	0.0000	0.4167	0.5882	0.7482	0.6586
SVM	0.5029	0.8800	0.3786	0.5875	0.6400	0.7316	0.6300
Adaboost	0.5923	0.7700	0.6214	0.6833	0.6696	0.7939	0.7597
XGBoost	0.6190	0.7800	0.6571	0.7083	0.6903	0.8203	0.7497
LightGBM	0.5655	0.9500	0.4786	0.6750	0.7090	0.8501	0.7806
AutoML	0.6234	0.9600	0.5857	0.7417	0.7559	0.9251	0.8712

Statistical Comparison of Model Performance: To quantitatively determine whether the superior predictive performance of the proposed AutoML framework was statistically significant, DeLong tests were performed to compare its ROC-AUC against each baseline model on both the cross-validated training set and the hold-out test set. As detailed in **Table 5**, on the independent test set, the AutoML model demonstrated a statistically significant improvement in discriminatory ability compared to all baseline models (all p< 0.001). Specifically, the difference in AUC between AutoML and the best-performing baseline model (LightGBM) was 0.0749 (95% CI: 0.0421 to 0.1078, p< 0.001). Similar significant advantages were consistently observed in the cross-validated training set comparisons (all p< 0.001), reinforcing the robustness of the performance gain attributable to the integrated two-stage optimization and automated pipeline.

**Table 5:** Statistical comparison of model performance using DeLong test.

Comparison (AutoML vs.)	Dataset	AUC difference (ΔAUC)	95% Confidence interval	Z statistic	P-value
Logistic Regression (LR)	Training Set	0.1674	(0.1342, 0.2006)	9.82	<0.001
	Test Set	0.1769	(0.1260, 0.2278)	6.78	<0.001
Support Vector Machine (SVM)	Training Set	0.1699	(0.1360, 0.2038)	9.90	<0.001
	Test Set	0.1935	(0.1441, 0.2429)	7.58	<0.001
AdaBoost	Training Set	0.0952	(0.0661, 0.1243)	6.45	<0.001
	Test Set	0.1312	(0.0843, 0.1781)	5.52	<0.001
XGBoost	Training Set	0.082	(0.0543, 0.1097)	5.86	<0.001
	Test Set	0.1048	(0.0581, 0.1515)	4.40	<0.001
LightGBM	Training Set	0.0362	(0.0181, 0.0543)	3.92	<0.001
	Test Set	0.0749	(0.0421, 0.1078)	4.48	<0.001

### 3.5 Interpretability analysis

#### 3.5.1 LASSO regression

**Figure 6:**
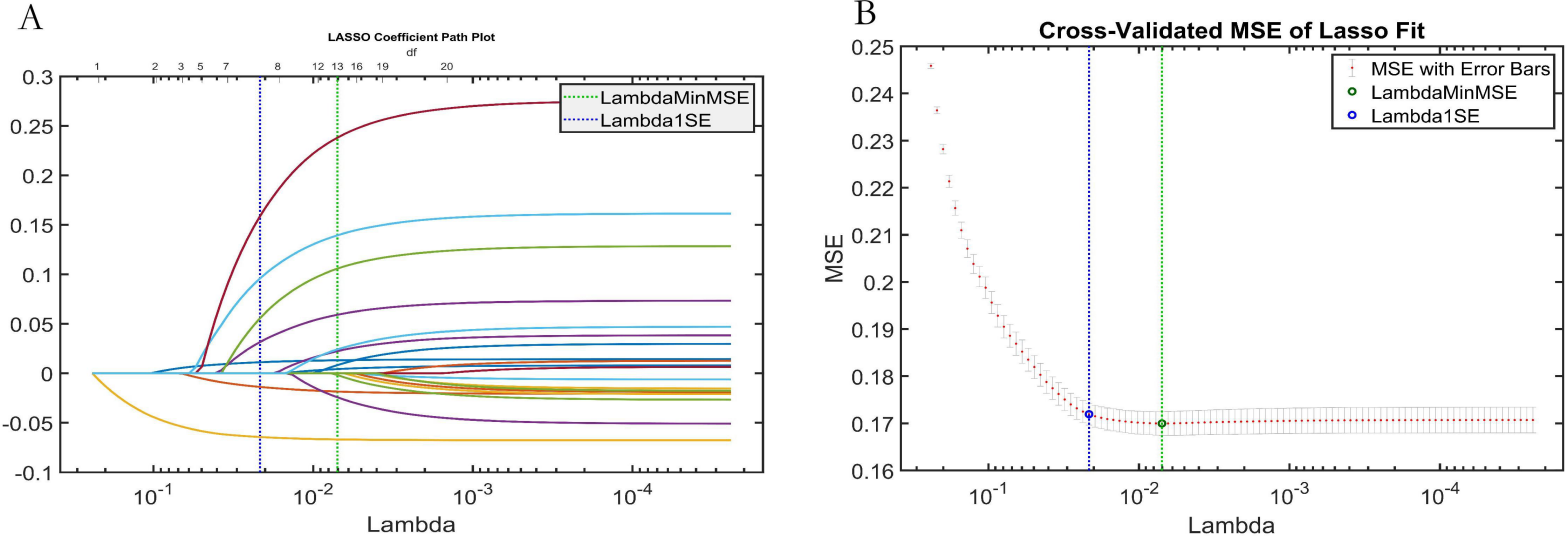
LASSO regression results. Note: **(A)** LASSO trajectory plot; **(B)** LASSO cross-validation fitting plot.

LASSO selected 7 features: Age, Diabetes, Preoperative retention, Catheter duration, Double-J stent duration, ALB, and Hospital stay (**Figures 6A, B**). Feature overlap with AutoML was 85.71% (6/7).

#### 3.5.2 SHAP analysis

**Figure 7:**
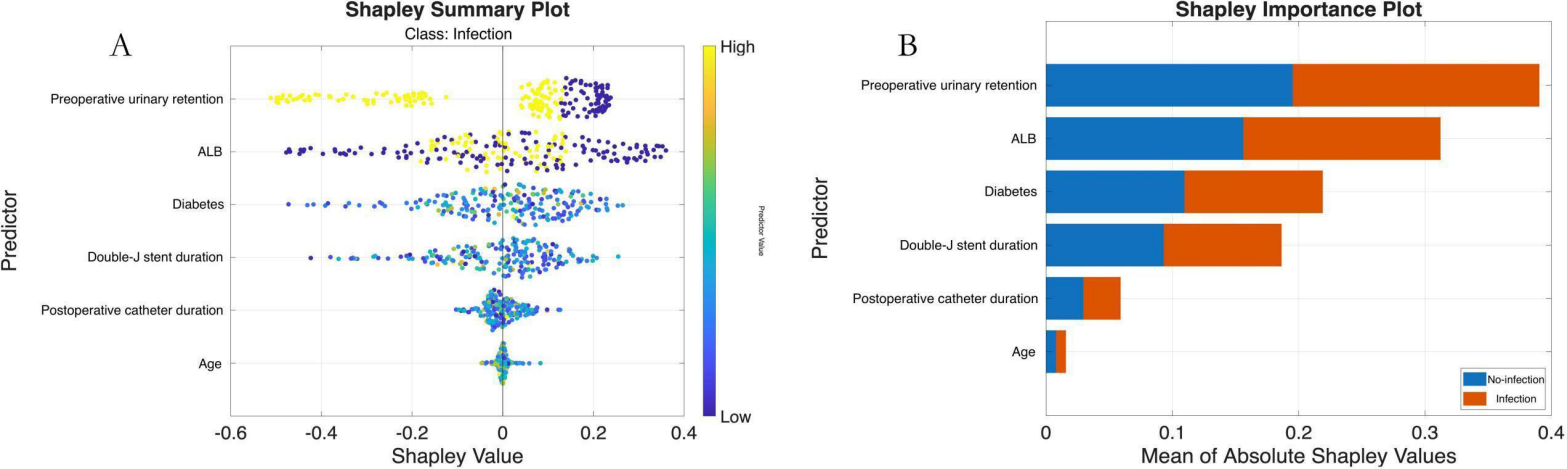
Comparison of overall SHAP values for key features. Note: **(A)** Sharpley summary plot; **(B)** Sharpley feature importance plot.

**Figure 8:**
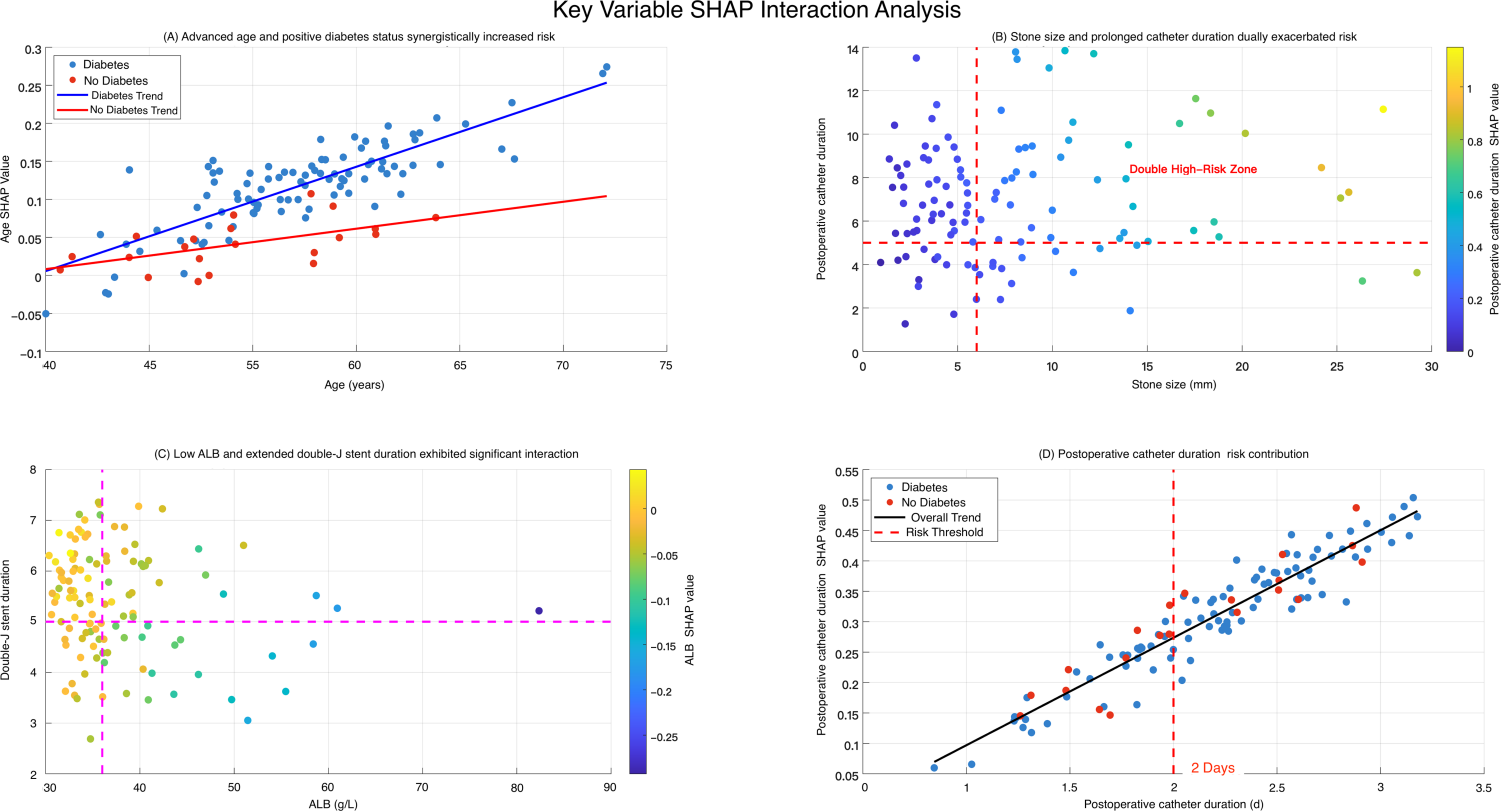
SHAP interaction analysis diagram for key features.

Based on the SHAP analysis results, the feature importance ranking was as follows: 1. preoperative urinary retention; 2. ALB; 3. diabetes; 4. double-J stent duration; 5. postoperative catheter duration; 6. age (**Figure 7**). The SHAP interaction analysis revealed (**Figure 8**): (A) Advanced age and positive diabetes status synergistically increased infection risk; (B) Stone size and prolonged catheter duration dually exacerbated infection risk; (C) Low ALB and extended double-J stent duration exhibited significant interaction; (D) Postoperative catheter duration >2 days significantly elevated risk.

### 3.6 Clinical decision support system

**Figure 9:**
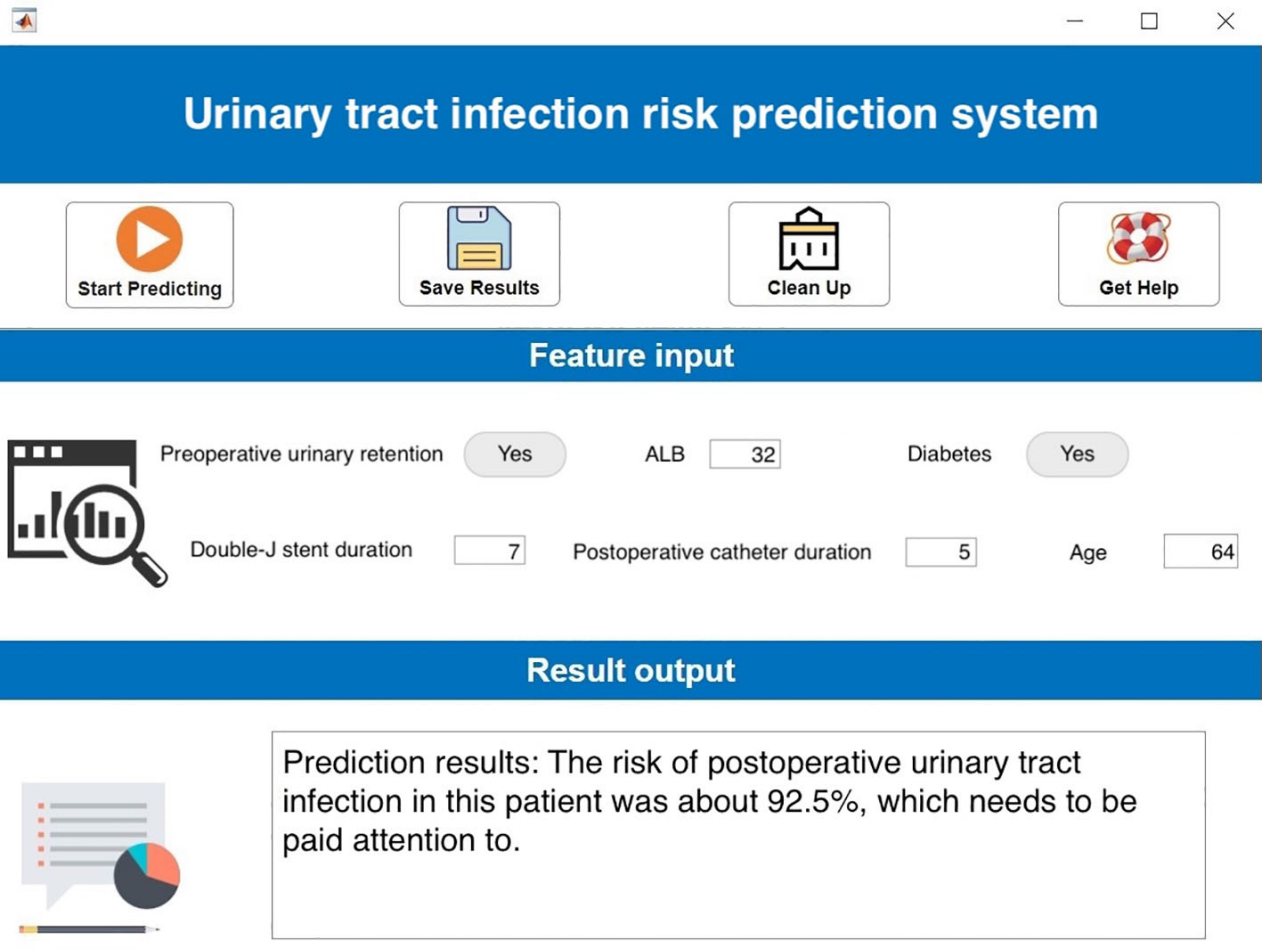
Clinical decision system demonstration.

To overcome barriers in AI adoption (e.g., programming skills), an intuitive visualization system was developed using MATLAB 2024a App Designer (**Figure 9**). Clinicians input key features to obtain real-time PO-UTI probability (%).

## 4 Discussion

This study systematically explored the risk prediction mechanism of postoperative urinary tract infection (PO-UTI) in ureteral stone patients by integrating automated machine learning (AutoML) and explainable artificial intelligence techniques. The AutoML framework was purposefully designed to optimize predictive accuracy rather than infer causal pathways, generating a robust classifier for PO-UTI risk stratification based solely on pattern recognition in heterogeneous clinical data. The ISequoiaOA framework significantly enhances stability over original SequoiaOA through two key modifications: Chaotic mapping initialization generates more diverse initial populations to escape local optima traps; Dynamic Lévy flight step size adaptively adjusts global exploration and local exploitation during convergence. These improvements were validated across all 12 CEC2022 benchmark functions, where ISequoiaOA exhibited smaller interquartile ranges and lower standard deviation in fitness values compared to its predecessor, confirming superior robustness against stochastic perturbations. Crucially, this stability directly translated to clinical modeling where feature selection sensitivity increased (AutoML vs. LASSO SHAP overlap), enabling reliable capture of critical nonlinear interactions like stone size-catheterization synergy that were missed by conventional methods.

Preoperative urinary retention was identified as the primary predictor, with its mechanism elucidated through three distinct pathological pathways: first, bladder overdistension impairs mucosal blood supply and reduces local oxygen partial pressure; second, residual urine serves as a liquid medium facilitating pathogen proliferation (El Husseini et al., 2024); and third, neurogenic damage to the bladder wall blunts normal urinary sensation (Coelho et al., 2023). Critically, our SHAP interpretability analysis identified serum albumin (ALB) and diabetes as the second and third most influential predictors of PO−UTI, substantiating the presence of a clinically meaningful metabolic−immune axis imbalance. This imbalance is mechanistically explained by a dual−hit process: chronic hyperglycemia in diabetic patients not only impairs neutrophil chemotaxis but also promotes type III fimbriae expression in *Escherichia coli*, thereby enhancing urothelial adherence (Schwartz et al., 2024); simultaneously, low serum albumin suppresses EGFR signaling pathway activity, which markedly delays urinary mucosal re−epithelialization and compromises the host’s primary physical barrier (Mohanty et al., 2022).Regarding device-related risks, the synergistic effect between double-J stent duration and postoperative catheter duration exhibited a critical threshold characteristic. SHAP interaction plots clearly indicated increased infection risk after >48 hours of catheterization, potentially linked to mature biofilm formation on catheter surfaces that drastically reduces cephalosporin permeability (Bull et al., 2025).

The pathogen distribution in this study revealed distinct characteristics. Among the 258 isolates from infected patients, gram-negative bacteria predominated (58.91%), with *Escherichia coli* (18.60%) and *Pseudomonas aeruginosa* (16.28%) constituting the main pathogens, aligning with typical urinary infection profiles (Kumar et al., 2021). Notably, gram-positive bacteria accounted for 37.21% of isolates—significantly higher than conventional UTI rates—with Staphylococcus aureus (13.95%) and Streptococcus haemolyticus (10.08%) being predominant (Massana Roquero et al., 2025). This deviation may stem from mucosal barrier disruption during surgery, facilitating the introduction of skin-colonizing bacteria (e.g., staphylococci). Fungal infections (Candida albicans, 3.88%) reflected dysbiosis risks from postoperative broad-spectrum antibiotic use (La Bella et al., 2025). The distribution pattern correlates critically with SHAP-identified predictors: 1) urinary stasis from preoperative retention favored E. coli proliferation, explaining its predominance (Miao et al., 2025); 2) the >48-hour catheterization threshold provided a key window for P. aeruginosa biofilm formation (Bull et al., 2025) (Miao et al., 2025); and 3) diabetic hyperglycemia potentially contributed to elevated gram-positive rates via enhanced pathogen adhesion (Schwartz et al., 2024) (Zou et al., 2024). Clinically, these findings highlight: 1) strict aseptic protocols during double-J stent placement due to iatrogenic pathogens; 2) initial empiric therapy prioritizing third-generation cephalosporins for ESBL-producing E. coli, balanced against fungal risk; and 3) mucosal injury from large stones facilitating skin flora invasion, partially explaining unexpected gram-positive prevalence (Massana Roquero et al., 2025) (Miao et al., 2025).

Compared with previous literature (Jalali et al., 2024; Cui et al., 2025; Zhou et al., 2025), this study achieved three major advances: 1) Development of ISequoiaOA metaheuristic algorithm framework improving feature selection sensitivity beyond LASSO; 2) First systematic application of SMOTE-ENN hybrid sampling to balance clinical data in PO-UTI prediction; and 3) Real-time individualized risk calculation via an interpretable clinical decision system that surpassed Charlson index accuracy. Particularly, the “stone-size vs. LASSO selection paradox” merits scrutiny: stone size (>6mm) demonstrated nonlinear synergy with prolonged catheterization (>5 days) in SHAP analysis—a critical interaction missed by LASSO regression. While both large stones (structural damage) and urinary retention (functional impairment) activate inflammation pathways (Kanlaya and Thongboonkerd, 2022; Tasian et al., 2025), LASSO’s L1 regularization favors univariate predictors, whereas AutoML-SHAP captured stone size’s exponential risk amplification with extended catheterization. This necessitates multidimensional alerts: enhanced catheter management in >6mm stone patients even without retention symptoms.

While our proposed AutoML model demonstrated high discriminative ability during cross-validation on the training set (ROC-AUC=0.9654, PR-AUC=0.9561), its performance on the independent test set showed a modest yet expected decrease (ROC-AUC=0.9251, PR-AUC=0.8712). This divergence aligns with typical machine learning behavior, where robust training performance may partially reflect over-optimization due to hyperparameter tuning within the training cohort. Critically, the test-set performance remains substantially superior to all baseline models (LR, SVM, AdaBoost, XGBoost, LightGBM) across both AUC metrics, confirming generalizable predictive utility. Additionally, the consistency between SHAP-derived biomarker importance (highlighting preoperative urinary retention, ALB, diabetes, and stent duration) and LASSO-selected clinical features underscores the biological plausibility of the model’s decision logic, mitigating concerns about pure data-driven overfitting. Future work will focus on refining regularization strategies to further bridge this performance gap in multicenter validation cohorts.

Limitations include: 1) The single-center retrospective nature of this study inherently limits the generalizability of our findings due to potential selection bias and the exclusion of specific high-risk populations, such as immunocompromised individuals. This design limitation underscores the critical need for future validation through multicenter prospective studies to ensure broader applicability; 2) Insufficient biomarker depth—overreliance on routine indicators (ALB, WBC) without novel inflammatory markers (suPAR, presepsin) or dynamic biofilm monitoring; and 3) Technical validation gaps: ISequoiaOA requires multicenter verification despite superior CEC2022 performance, and the decision system lacks prospective efficacy trials. Despite rigorous validation via LASSO-SHAP concordance, the observational nature of this analysis precludes causative interpretations of identified risk factors; 4) While our MATLAB-based decision tool proves the CDSS prototype’s technical feasibility, critical implementation barriers remain unaddressed: Interoperability with hospital electronic health record (EHR) systems requires HL7/FHIR-compliant API development for seamless clinical deployment; Formal usability studies evaluating clinician workflow integration and interface intuitiveness are pending; Prospective validation in real-world settings is essential to assess actual impact on clinical outcomes—a gap common among initial AutoML implementations.

Future directions include: 1) Multicenter prospective cohorts incorporating high-risk subgroups with bedside biofilm monitoring; 2) Enhanced time-series modeling to quantify dynamic intervention impacts (e.g., antibiotic prophylaxis efficacy, catheter removal timing); 3) Multi-omics-based risk systems integrating novel inflammatory markers and urinary pathogen metagenomics; and 4) EHR-integrated closed-loop management with stepwise interventions and cluster-randomized trials to assess antibiotic-reduction benefits; To transition from prototype to practice, we will: Develop HL7-compatible APIs for EHR integration using Fast Healthcare Interoperability Resources (FHIR) standards; Conduct structured usability trials (SUS scores, task-completion analytics) with urology teams; Initiate a multicenter prospective cohort to measure CDSS-driven reductions in PO-UTI incidence and antibiotic misuse.

## 5 Conclusion

This study pioneered dynamic PO-UTI prediction by integrating AutoML with explainable AI. SHAP analysis quantified critical interaction thresholds and revealed the centrality of preoperative urinary retention-ALB-diabetes “triangular network”. These advances transition urological infection management from empirical to data-mechanism dual-drive paradigms. While our decision system demonstrates robust predictive performance, the clinical translation of these findings remains constrained by the single-center retrospective design. External validation using diverse, multi-institutional cohorts is therefore paramount to confirm generalizability before widespread clinical implementation.

## Data Availability

The raw data supporting the conclusions of this article will be made available by the authors, without undue reservation.
