# Supplementary material for: Development of a prediction model for postoperative urinary tract infection in ureteral stone patients based on automated machine learning models
Source: Front Physiol. 2026 May 26;17:1768212. doi: 10.3389/fphys.2026.1768212 (PMC13246413; doi:10.3389/fphys.2026.1768212)
Supplement: Supplementary file 1 [file Table1.docx]

**PSEUDOCODE: Two-phase AutoML Framework**

% Input: Training data (features, labels)

% Output: Optimal feature subset, optimal model and hyperparameters

% --- Phase 1: Feature Selection via ISequoiaOA (Discrete Space Optimization) ---

function selectedFeatures = phase1_FeatureSelection(features, labels)

% Initialization

Initialize population using chaotic mapping (each individual as a binary feature mask);

Define fitness function: ROC-AUC based on 5-fold cross-validation;

% ISequoiaOA Optimization Loop

while convergence NOT met (e.g., no improvement in best fitness for 50 generations)

for each individual in the population

% Exploration & Exploitation via Dynamic Lévy Flight

Calculate movement step size;

Generate new candidate solution;

% Self-regulation Mechanism (inspired by sequoia competition)

Apply competitive pressure based on fitness ranking;

Update individual position;

end

Evaluate population fitness;

Update global best solution;

end

selectedFeatures = global_best_binary_mask; % Output indices of selected features

end

% --- Phase 2: Hyperparameter Tuning on Selected Features (Continuous Space Optimization) ---

function [tunedModel, bestHyperparams] = phase2_HyperparameterTuning(features_selected, labels)

% Define hyperparameter search space (example: LightGBM)

searchSpace = struct(...

'LearningRate', loguniform(0.001, 0.1), ...

'NumLeaves', uniformInteger(20, 150), ...

'RegLambda', loguniform(1e-3, 10) ...

);

% Initialization & Optimization Loop (Reusing ISequoiaOA core in continuous space)

Initialize hyperparameter population;

while convergence NOT met

Update population positions (using chaotic dynamics & Lévy flight);

for each hyperparameter set

Train a temporary model on the feature subset;

Calculate validation ROC-AUC via nested CV as fitness;

end

Update global best hyperparameter set;

end

% Output

bestHyperparams = global_best_hyperparameters;

tunedModel = trainFinalModel(features_selected, labels, bestHyperparams);

end

% --- Main Workflow ---

% 1. Data Preparation & Splitting

[trainData, testData, trainLabels, testLabels] = splitData(allData, 0.8);

% 2. Handle Class Imbalance (SMOTE-ENN)

trainDataBalanced = apply_SMOTE_ENN(trainData, trainLabels);

% 3. Two-phase Automated Modeling

featMask = phase1_FeatureSelection(trainDataBalanced, trainLabels); % Phase 1

trainDataReduced = trainDataBalanced(:, featMask);

[finalModel, optimalHyperparams] = phase2_HyperparameterTuning(trainDataReduced, trainLabels); % Phase 2

% 4. Evaluation on Hold-out Test Set

testDataReduced = testData(:, featMask);

predictions = finalModel.predict(testDataReduced);

performance = evaluateModel(predictions, testLabels);
